# The role of the extracellular matrix in primary myelofibrosis

**DOI:** 10.1038/bcj.2017.6

**Published:** 2017-02-03

**Authors:** O Leiva, S K Ng, S Chitalia, A Balduini, S Matsuura, K Ravid

**Affiliations:** 1 Department of Medicine and Whitaker Cardiovascular Institute, Boston University School of Medicine, Boston, MA, USA; 2 Department of Molecular Medicine, University of Pavia, Pavia, Italy; 3 Laboratory of Biotechnology, IRCCS San Matteo Foundation, Pavia, Italy

## Abstract

Primary myelofibrosis (PMF) is a myeloproliferative neoplasm that arises from clonal proliferation of hematopoietic stem cells and leads to progressive bone marrow (BM) fibrosis. While cellular mutations involved in the development of PMF have been heavily investigated, noteworthy is the important role the extracellular matrix (ECM) plays in the progression of BM fibrosis. This review surveys ECM proteins contributors of PMF, and highlights how better understanding of the control of the ECM within the BM niche may lead to combined therapeutic options in PMF.

## Primary myelofibrosis, its clinical manifestations and common mutations

Chronic myeloproliferative neoplasms (MPNs) are a heterogeneous group of disorders arising from clonal proliferation of hematopoietic stem cells. Primary myelofibrosis (PMF), polycythemia vera and essential thrombocythemia (ET) are the main Philadelphia chromosome negative MPNs.^1^ The clinical presentation of each of these disorders varies, although all have the potential for leukemic transformation and thrombohemorrhagic events.

PMF is described as either pre-fibrotic PMF (prePMF) or overt PMF according to the 2016 WHO diagnostic criteria. Compared to the 2008 WHO diagnostic criteria, the 2016 WHO criteria make a distinction between prePMF and overt PMF (Table 1). This distinction is especially important because prePMF can present similarly and be mistaken for ET. Making the correct diagnosis is important given the poorer prognosis, increased mortality and leukemic transformation rate for prePMF compared to ET.^2, 3^

Although a substantial number of patients with polycythemia vera and ET are asymptomatic at presentation, patients with PMF commonly complain of fatigue and symptoms due to splenomegaly.^4, 5^ Hallmarks of PMF include expansion of the megakaryocytic lineage, bone marrow (BM) fibrosis and extramedullary hematopoesis (EMH), which occurs predominantly in the liver and spleen, but can manifest anywhere.^6, 7^ Patients with PMF can also have portal hypertension primarily through increased splenic blood flow, hepatic EMH and sinusoidal fibrosis.^8^ Overall survival for PMF is significantly lower than for polycythemia vera and ET, and one study showed PMF patients were also more likely to have leukemic transformation.^9^

Several somatic mutations have been identified in PMF, including in *JAK2, MPL, CALR* and several other genes. Janus kinase 2 (JAK2) is a cytoplasmic tyrosine kinase engaged with numerous intracellular signaling pathways involving receptors for erythropoetin, thrombopoetin, interleukin-3, granulocyte colony-stimulating factor and granulocyte–macrophage colony-stimulating factor.^10^ A single acquired somatic point mutation at V617F in JAK2 causes MPN in patients.^11, 12^ JAK2V617F is found in 95% polycythemia vera patients and detected in ~60% of ET and PMF patients.^9^ The JAK2V617F mutation affects the pseudokinase domain of *JAK2* and makes JAK2 constitutively active.^13^

Another common mutation in PMF is in the Calreticulin (*CALR*) gene, which accounts for 25% of PMF patients.^14, 15^ *CALR* functions as an ER chaperone and its mutation activates both the thrombopoietin receptor, c-mpl and JAK2.^16^ Patients with PMF and CALR mutations are younger and have lower risk of death than their JAK2 and MPL-mutated counterparts, despite their higher platelet count.^17^ Another identified mutation that leads to 5% of PMF cases is due to a somatic gain-of-function at amino acid residues W515 (W515K/L) and S505 mutation in the transmembrane domain of c-mpl, a receptor that activates downstream JAK/STAT signaling.^18^

The prognosis of patients with PMF is generally poor, but depending on the mutations involved it appears that survival and adverse outcomes can vary. As mentioned before, JAK2, CALR and c-mpl are driver mutations that account for 90% of PMF cases, while 10% can be viewed as ‘triple negative'. One study found differences in median survival in patients with PMF that either had JAK2, CALR, c-mpl mutations or were triple negative. Patients with CALR-mutated PMF have a more favorable prognosis, while triple negative PMF patients have the worst prognosis (median survival in one study of CALR-mutated PMF is 15.9 years vs 2.3 years in triple negative PMF).^9, 19^ Mutations in IDH1/2, SRSF2 and ASXL1 in PMF were shown to have an increase risk of leukemic transformation.^20^ In one study, patients with CALR mutations and no ASXL1 mutation (CALR^+^ASXL1^−^) had the longest survival, while CALR^-^ASXKL^+^ had the shortest survival (median survival of 10.4 years vs 2.3 years respectively).^21^ Interestingly, ASXL1, EZH2 and IDH1/2 have been shown to play a role in chromatin structure, suggesting that epigenetic dysregulation may play a role in PMF progression and leukemic transformation.^22^

## The bone marrow niche and extracellular matrix

### The BM niche

The BM is a spongy tissue within the central cavity of several bones of the body.^23^ The BM space is evenly occupied by sinusoids. The endosteal surface of the bones and cells constitute the stem cell niche in which the hematopoietic stem cells (HSCs) reside and differentiate to different lineages.^24, 25, 26, 27^ The BM niche is separated into two compartments. The first compartment is the osteoblastic niche found near the endosteum and the second compartment is the vascular niche near the sinusoids.^28^ These two niches consist of different cell types such as adipocytes, osteoblasts and smooth muscle cells, Schwann cells, reticular cells, endothelial cells and hematopoietic cells.^14, 25^ However, there is no distinct separation between the two niches as HSCs can move freely and can receive inputs from the two compartments simultaneously.^29^ The niches also contain stromal cells and unique extracellular matrix (ECM) components that support stem cells by HSCs interaction with other cells through cell surface receptors, gap junctions and soluble factors.^30^ That is, the molecular crosstalk between HSCs and the cellular constituents of these niches determine the balance between HSC self-renewal and differentiation.^31^

### The osteoblastic niche

The osteoblastic niche is composed of several types of cells that aid in the maintenance of HSC. Expansion of HSCs by osteoblast factors has been shown *in vitro* (via production of granulocyte colony-stimulating factor) as well as *in vivo*, suggesting that osteoblasts play an important role in HSC maintenance.^24, 32, 33, 34^ However, activated osteoblasts have also been shown to produce osteopontin and angiopoietin-1, which limit HSC expansion and contribute to their quiescence state.^28^ N-cadherin at the niche helps HSCs to adhere to the osteoblastic niche through tight cell–cell interactions, and overexpression of N-cadherin promotes quiescence.^30^ Osteoclasts also appear to be important in the osteoblastic niche by releasing calcium during bone resorption. HSCs were found to express calcium-sensing receptors. Mice deficient in calcium-sensing receptors were found to have hypoplastic BM with decreased localization of HSCs in the osteoblastic niche but had normal number of HSCs in the fetal liver.^35^ Interactions between the niche and hematopoietic cells are reciprocal. For instance, megakaryocytes (MKs) overexpressing BMP 2, 4 and 6 at the endosteum have been shown to stimulate osteoblasts.^36^ Hence, HSCs and its cell lineages are involved in bone formation and activities within the niche.^37^ Non-bone components are also important in the maintenance of HSCs within the osteoblastic niche. CXCL12 is a chemokine that plays an important role in the maintenance and quiescence of HSCs through its receptor CXCR4 in both the osteoblastic and vascular niches.^38^ Interestingly, the sympathetic nervous system appears to modulate CXCL12 expression in a circadian pattern through the release of norepinephrine which downregulates CXCL12. Circulating HSCs were found to peak at five hours after the initiation of light with ablation of sympathetic nerve fibers decreasing the numbers of circulating HSCs in mice.^39, 40^ Sympathetic nerve fibers also appear to have an important role in BM regeneration as evidenced by impaired HSC recovery after treatment with cisplatin (a neurotoxic chemotherapeutic drug) in mice.^41^

### The vascular niche

The vasculature niche is located in the perivascular space and helps to activate HSCs to differentiate into other blood lineage.^42^ The vascular niche is located around small sinusoidal blood vessels associated with various stromal elements and together with fibronectin, type IV collagen and laminin at the ECM helps to regulate HSC differentiation and ultimately mobilization of differentiated blood cells to the peripheral circulation.^26, 30^ Endothelial cells at the vascular niche secrete E-selectin and promote HSC proliferation.^37^ The walls of venous sinusoids consists of a layer of endothelial cells which acts as a conduit for mature blood cells and platelets to enter the blood stream from the BM compartment.^43^ Simultaneously, blood vessels provide a conduit for distal systemic signals, such as inflammatory and other circulatory cells into the niche.^44^ Besides the effect of cytokine and chemokine, a hypoxic environment in the osteoblastic niche helps to maintain HSCs in a quiescent state, while a more oxygen rich environment in the vascular niche allows HSCs to proliferate and differentiate.^42^

## Disruption of BM niches in PMF

Abnormal interactions between the HSCs and its microenvironment in the BM can affect PMF disease progression.^45^ HSCs harboring mutated genes such as JAK2V16F can alter the niche to favor their clonal expansion at the expense of normal HSCs.^14^ One example of this is that JAK2V617F mutant HSCs secrete interleukin-1β, which activates the apoptotic pathway in mesenchymal and Schwann cells. This will, in turn, affect the survival rate of normal HSCs via disruption of the interactions between the mesenchymal cells, and injury to sympathetic nerves innervating the BM. Mice with JAK2V617F MPN had decreased numbers of Schwann cells and MPN progression was slowed by the administration of β3 adrenergic agonists that compensated for the neuropathy seen in the BM of these mice.^46^ In addition, mutated MKs release excessive amount of fibrotic factors, which activate mesenchymal cells, leading to MF.^14^ In one study of PMF patients, several genes that may be related to the maintenance of the BM niche homeostasis were found to be dysregulated. Genes such as hypoxia inducible factor 1A, CXCR4 and PAX5 were found to be downregulated in BM mononuclear cells from patients with PMF, while cyclooxygenase 2 was found to be significantly upregulated.^47^ Furthermore, malignant stem cells from a mouse model of MPN appear to modify the osteoblastic niche to benefit their survival at the expense of the survival of non-malignant HSC by causing mesenchymal stromal cells to overproduce osteoblastic lineage cells that promote inflammation and MF.^48^ Furthermore, malignant stem cells have also been shown to produce high levels of lipocalin2, which was shown to increase proliferation of PMF HSC while decreasing the numbers of normal HSC.^49^ Stromal cells in BM undergo changes associated with myeloproliferation which include excessive ECM deposition leading to fibrosis, neoangiogenesis and osteosclerosis.^50, 51, 52^ The JAK2V617F mutation has not only been shown in HSC and myeloid cells, but also in endothelial cells in patients with PMF and other MPNs.^53, 54^ One study suggests that JAK2V617F mutated endothelial cells in the vascular niche could promote malignant stem cell proliferation.^55^ These changes may eventually disrupt normal HSC niches and result in the establishment of EMH.^56, 57^ Although advances have been made in the understanding of how the BM niches are altered in MPNs and PMF in particular, our current understanding is incomplete.

## Extracellular matrix

The ECM is a three-dimensional, non-cellular structure providing physical support for tissue integrity and elasticity.^58^ It is comprised of various matrix proteins, such as collagens, laminin, fibronectin, vitronectin and fibrinogen as well as soluble proteins, including cytokines and chemokines.^59^ These components define the biochemical and biomechanical properties of the ECM and are able to influence the attachment of cells to the ECM and directly affect their biological functions, such as cell division, differentiation, tissue polarity and cell migration.^30^ At the same time, cells can sense ECM compositions and transmit appropriate signals at the adhesion sites.^30^ The adhesion interaction requires integrins and signaling pathways, including Ras/MAPK, PI3K/Akt, RhoA/ROCK, Wnt/β-catenin and TGF-β that link the actomyosin cytoskeleton with the ECM.^30^ At the BM niche, the ECM provides the microenvironment for HSCs to maintain a quiescence stage or to undergo differentiate to form various progenitors.^60, 61^

MKs differentiation and proplatelet formation depend on the stiffness of the matrix. One study found that MKs cultured on a methylcellulose hydrogel media that mimics BM stiffness exhibited higher ploidy levels than MKs cultured on liquid media. Furthermore, MKs cultured on the stiffer media produced twice as many proplatelets when placed in liquid media than those that were cultured in the liquid media.^62^ In a study looking at *ex vivo* platelet production on a three-dimensional silk BM niche, the authors found that low and medium stiffness silk film supported a higher percentage of long and branched proplatelets compared to high stiffness silk film, although adhesion was not different, suggesting that increased ECM stiffness may reduce platelet production.^23^ Interestingly, MKs on silk films entrapped with type I collagen had a significantly reduced proplatelet formation as compared to MKs on silk films entrapped with fibrinogen.^23^ Another study also showed that type I collagen is a negative regulator of proplatelet formation through activation of the integrinα2β1/Rho/ROCK axis.^63^ It has been noted by clinicians that fibrotic tissues are usually stiff and enriched with ECM components as compared to healthy tissues.^64, 65^ Furthermore, the level of tissue stiffness can be used as a predictive indicator for disease stage.^66^ Hence, there is a correlation between stiffness of the matrix and fibrosis, which may contribute to the clinical manifestations seen in PMF (thrombocytopenia, EMH).

**Figure 1:**
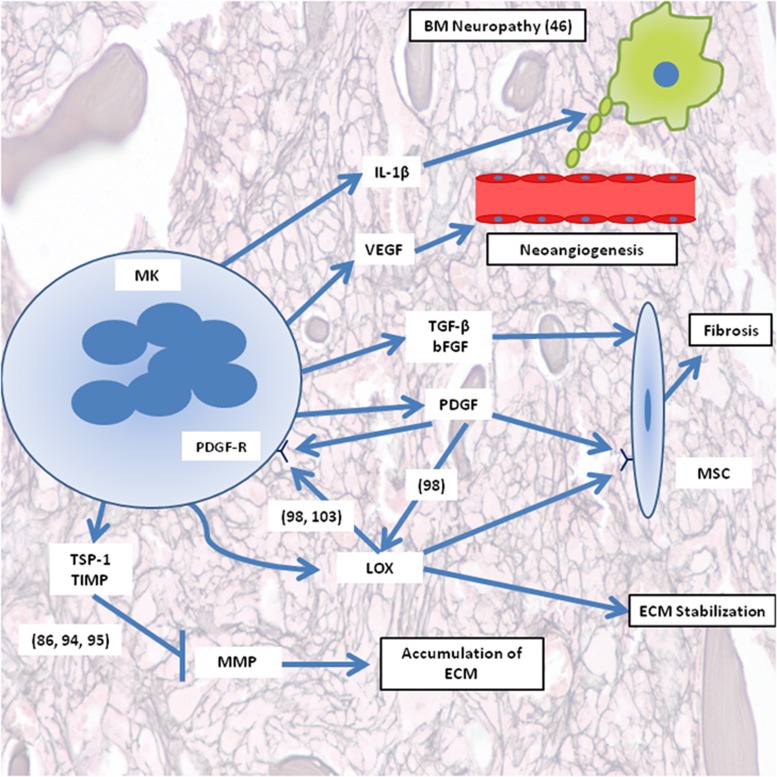
Schematic presentation of major components in ECM derived from MKs and involved in PMF progression. The parentheses include reference numbers corresponding to the illustrated pathway. BM, bone marrow; bFGF, basic fibroblast growth factor; ECM, extracellular matrix; IL-1β, interleukin-1 beta; LOX, lysyl oxidase; MK, megakaryocyte; MMP, matrix metalloproteinase; MSC, mesenchymal stromal cell; PDGF-R, PDGF receptor; TGF-β, transforming factor beta; TIMP, tissue inhibitor of metalloproteinases; TSP-1, thrombospondin-1; PDGF, platelet derived growth factor; VEGF, vascular endothelial growth factor.

In PMF there is progressive deposition of ECM components in the BM, and PMF patients have more ECM materials than healthy population.^67, 68, 69^ MKs are known to secrete ECM components such as fibronectin, laminin and type IV collagen, which contribute to MK development and ECM homeostasis, therefore, dysfunctional MKs can potentially play a role in pathogenesis of PMF by secreting excessive amount of ECM components.^70, 71^ Below we summarize the components of the ECM and their role in PMF (Figure 1).

## Structural proteins of the ECM and their relevance to and role in PMF

### Collagens

MKs secrete various types of collagens found in the BM ECM and their abundance differ between the BM osteoblast and vascular niches.^23^ For instance, in the osteoblastic niche, collagen I is the most abundant component, and binding of MKs to collagen through integrin inhibits proplatelet formation.^72, 73^ In addition, collagen I creates an environment for HSCs to differentiate through the megakaryocytic lineage but inhibit pro-platelet formation and release is inhibited through adhesion to type I collagen via activation of the Rho/ROCK signaling cascade.^73, 74^ In the vascular niche, MKs interact with collagen IV at the microenvironment which allow MKs to mature and form proplatelet.^26^ Beside differences in abundance at the osteoblastic and vascular niche, different collagens are localized in various regions of the ECM niche. Type I, III and V collagens form fibers in the ECM, which provides elasticity and flexibility to the matrix.^75, 76^ Interestingly, unlike type I collagen, type III and V collagens were found to support proplatelet formation *in vitro*.^70^ Type IV, VIII and XVIII collagens are expressed directly beneath the endothelial cells in the basement membrane and are commonly associated with laminin.^75^ BM fibroblast activated by factors released from PMF MKs, such as TGF-β, upregulate the expression of collagen, leading to augmented level deposition of this protein in the ECM in PMF patients.^77^ MKs from PMF patients also have increased expression of type III and type IV collagens as compared to MKs derived from healthy controls.^71^ Interestingly, in patients with PMF, as the grade of fibrosis increases the platelet count drops, which may be partly explained by changes in the ECM in later stages of the disease.^78^

### Glycoproteins

#### Fibronectin

Fibronectin is involved in various cellular interactions in the ECM and is important in cell adhesion, migration and growth and differentiation.^79^ Human MKs expresses and secrete cellular fibronectin which is involved in MKs maturation, platelet extension and subsequent release.^80^ Fibronectin is abundantly found in BM niche and it is able to stimulate HSCs and MKs proliferation via fibronectin receptors VLA-4 and VLA-5.^26^ Fibronectin assembly in the matrix can be influence by integrin activation and contractility of the MKs cytoskeleton.^80^ Fibronectin surrounds cells and provide structural scaffolding for tissues.^58^ *In vitro*, fibronectin cause a threefold increase in mouse HSCs proliferation with a subsequent higher MK output when compared to control treated with thrombopoetin only, suggesting the importance of fibronectin in MKs development.^70^

In patients with pre-fibrotic MPNs, mesenchymal stromal cells were found to secrete more fibronectin than in controls. The amount of fibronectin expression was correlated to reduced hemoglobin levels in patients with MPN in the absence of reticulin fibrosis.^81^ In another study, patients with PMF were found to have elevated mesenchymal stromal cell expression of fibronectin compared to patients with ET and controls.^82^ One study showed that fibronectin can activate monocytes in patients with MF via increased monocytic production of substance P, a proinflammatory cytokine.^83^

#### Thrombospondin

Thrombospondin (TSP) was identified in thrombin-stimulated platelets, and is also expressed by a variety of cells, such as endothelial cells, fibroblasts and smooth muscle cells.^84^ TSPs are matricellular proteins that interact with cell surface receptors and with components of the ECM, thereby mediating cell–matrix interactions.^85^ Currently, there are 5 known members of TSP1 family. TSP-1, TSP-2, TSP-3, TSP-4 and TSP-5. TSP-1 and TSP-2 are better understood as compared to the other 3 members.^86^ TSP are involved in MK development and platelet function.^87^

TSP-1 is also an activator of latent TGF-β1, which is an inducer of fibroblast activation and matrix synthesis in the fibrotic response and in PMF.^88, 89^ TSP-1 was significantly overexpressed in all stages of PMF independently of the degree of MF, when compared to controls. Individual follow-up biopsies showed involvement of TSP-1 during progressive MF. TSP-2 is only strongly expressed in 40% of cases with advanced MF. Interestingly, MKs and interstitial proplatelet formations were shown to be the relevant source for TSP-1 in PMF. In addition, TSP-1 inhibits the activity of matrix metalloproteinases (MMPs), which are primarily involved in proteolysis of collagens and other matrix components, which allows the accumulation of ECM.^86^

#### Osteonectin

Osteonectin (also known as SPARC) is an adhesive calcium-binding ECM glycoprotein that binds various ECM components such as TSP-1 and fibrillar collagens.^90^ In adults, osteonectin is expressed during processes requiring ECM turnover such as wound healing and tumor progression.^90^ Osteonectin prevents cell spreading *in vitro* suggesting they play an important role in cellular proliferation and migration.^91^ High levels of tissue osteonectin has been associated with reduced collagen type IV deposition.^92^

Interestingly in healthy individuals and in myeloid neoplasms without associated stromal changes, osteonectin expression was confined to MKs.^50^ In contrast, in cases with significant stromal changes (such as PMF), osteonectin reactivity extends to stromal cells. Hence, osteonectin is part of BM response to myeloproliferation. That is, the expression level of osteonectin in BM stromal cells correlates with the degree of stromal changes and correspond to the severity of PMF.^50^ This is further shown in osteonectin knockout mice in which there is impairment in BM fibrosis.^50^

## Modifiers of ECM structure and function and their relevance to and role in PMF

### Matrix MMPs

MMPs are a family of zinc-dependent endopeptidases and function in remodeling the ECM by its ability to degrade and cleave ECM components with wide substrate specificities.^93^ For instance, MMP2 and MMP9 are effective in degrading collagen and gelatine structures in the ECM.^93^ In addition, by producing MMP9, mature MKs are able to free themselves from the BM matrix at the osteoblastic niche and travel to the vascular niche. In patients with PMF, MMPs are downregulated while tissue inhibitors of MMPs (TIMP) are increased.^94, 95^ This leads to decreased degradation and increased accumulation of ECM components.

### Lysyl oxidase

LOX is a copper-dependent amine oxidase that catalyzes oxidative deamination of lysine and hydroxylysine residues on collagen and elastin precursors, leading to crosslinking within these proteins.^96^ The crosslinking results in a dense ECM with altered elasticity.^97^ LOX is highly expressed in proliferating, low ploidy MKs, but its expression decreased dramatically in mature, higher ploidy mouse MKs.^98^ Following this observation, the Ravid laboratory was also the first to identify LOX as regulator of BM fibrosis in a mouse models of PMF.^98^ This link was demonstrated in a GATA-1^low^ mouse model where LOX was found to be abundantly expressed within abnormally high levels of low ploidy MKs coupled with an extensively fibrotic ECM.^98, 99^ Importantly, administration of β-aminopropionitrile (a LOX inhibitor) to the GATA-1^low^ mice inhibited the progression of MF.^98^ Similarly, LOX was reported to be upregulated in human PMF cells and plasma.^100^

Subsequently, it has been noted that abnormalities in MK expansion and proliferation are associated with increased levels of extracellular platelet derived growth factor (PDGF) and TGF-ß1, which can lead to fibrosis.^101, 102^ PDGF, TGF-β1 and the cytokine interleukin-1β are able to increase LOX and collagen expression, all of which have been found to be elevated in PMF.^101, 102^ LOX has also been shown to oxidize and activate PDGF receptor and LOX activity appears to be important for PDGF-mediated MK expansion.^98, 103^ Hence, there appears to be positive feedback in which LOX is highly secreted from abnormal MKs, and soluble factors secreted by these MKs can further increase LOX level, which will enhance the fibrotic phenotype (Figure 1).

### ECM bound growth and secreted factors

The ECM is maintained by several cytokines and growth factors. Dysfunctional HSCs and MKs have abnormal production and release of several cytokines and chemokines, which are associated with fibrosis and enhanced survival and proliferation of mutant HSCs, thus contributing to ECM disruption in PMF.^23, 104, 105, 106, 107^ Inflammatory cytokines have been found to be elevated in PMF regardless of the mutational status, including interleukin-1β and tumor necrosis factor alpha. Both of these pro-inflammatory cytokines have been shown to enhance mutant HSC proliferation and survival *in vitro*.^108^ Other cytokines released by malignant HSCs are profibrotic, such as transforming growth factor beta1 (TGF-β1), basic fibroblast growth factor, and PDGF, and angiogenic factors such as vascular endothelial growth factor (VEGF).^23^

### Transforming growth factor beta (TGF-β)

TGF-β1 plays a key role in regulation of genes involved in the synthesis of the ECM components and of ECM-degrading enzymes.^58^ For instance, TGF-β1 enhances the production of types I, III and type IV collagen and fibronectin, as well as increases the synthesis of TIMP.^94, 109^ There is also a strong correlation between MK release of TGF-β1 and its activity, resulting in a dose-dependent increase of ECM component synthesis.^110^ In the ECM, reactive oxygen species activate a number of proteases such as MMPs and TSP1, which can digest and convert the latent TGF-β to its active form.^111, 112^

TGF-β1 is involved in the pathophysiology of PMF and is a strong inducer of fibrosis.^113^ Not surprising, high TGF-β concentration is found in PMF BM and is correlated to BM fibrosis *in vivo*.^89^ In PMF, TGF-β1 affects ECM biosynthesis by decreasing the amount of MMP and increasing the synthesis of TIMPs, particularly of TIMP-1.^114^ TGF-β leads to an increase in production of types I, III, IV and V collagens, and to overexpression of fibronectin in advanced stage of the disease, which further accelerate ECM accumulation.^109^ MKs and platelets are the main source of TGF-β in PMF with intraplatelet levels being 2–3 times higher in PMF patients compared to healthy controls.^115, 116, 117^

### Platelet derived growth factor and vascular endothelial growth factor

MKs are an important source of PDGF and VEGF, which contribute to MK role in the development of BM fibrosis and production of collagen.^23^ PDGF participates in BM fibrogenesis in PMF through its role in the proliferation and activation of medullary fibroblasts.^59^ VEGF functions by binding to its receptor VEGFR1 to promote MK maturation and migration from osteoblastic niche to the vascular niche where proplatelets formation and platelet release occur.^118^

### Current treatment of PMF

Current management of PMF is primarily palliative and aimed at relieving symptoms of (anemia, splenomegaly, constitutional symptoms and bone pain). Only allogenic hematopoietic cell transplant is curative although few patients are eligible for this treatment.^119^

Hydroxyurea (HU) is a medication commonly used to treat symptomatic PMF and has been shown to significantly improve bone pain, constitutional symptoms and splenomegaly.^120^ Interestingly, response to HU may be related to the presence of JAK2V617F mutation in PMF with those harboring the mutation more likely to respond to HU.^121^ Anemia can be treated in PMF with red blood cell transfusions (although frequent transfusions can put patients at risk for iron overload), androgens and danazol. Thalidomide or lenalidomide with prednisone can be used for persistent anemia.^120, 122, 123^ Importantly, none of the aforementioned palliative therapies have been convincingly shown to decrease BM fibrosis.^122^

JAK1 and 2 inhibitors have been introduced as treatment of PMF, the only Food and Drug Administration (FDA) approved drug being ruxolitinib. In the phase 3 COMFORT I and II trials, ruxolitinib was shown to be more effective than best available therapy for reduction of splenomegaly and constitutional symptoms although it was associated with higher rates of anemia and cytopenias. Ruxolitinib also appears to reduce BM fibrosis in some patients.^124, 125^ Although the COMFORT I and II trials failed to show a survival advantage in ruxolitinib, long-term follow-up studies from COMFORT trials suggest a modest decrease in mortality in patients with intermediate-2 and high-risk disease.^124, 126, 127, 128^ Although ruxolitinib is the only non-allo HCT treatment currently available that may improve survival, the benefits appear to be modest and leave a lot to be desired in the treatment of PMF.

### Newer experimental therapeutics for PMF

Given the poor prognosis of PMF and limitations of current treatment options, novel drugs are needed to improve the quality of life and survival of patients. One such novel drug is the telomerase inhibitor imetelstat. A recent pilot study looked at 33 patients with intermediate-2 or high-risk MF and showed that four patients had complete response and three had partial response to imetelstat. All four patients with complete response had reversal of BM fibrosis with three having molecular remission. The study also showed symptomatic spleen reduction in 35% of patients and 31% of transfusion-dependent patients before the study became transfusion-independent for at least 3 months. Myelosupression was a common adverse requiring protocol-mandated dose reduction. Interestingly, response rates were seen in 27% of patients with JAK2 mutation and 0% seen in those without.^129^ More long-term studies with larger cohort are needed but imetelstat remains a promising prospective drug.

MKs in PMF have an impaired ability to polypoidize, yielding another potential target for therapy. Aurora kinase A (AURKA) appears to be important in maturation and polypoidization of MKs and studies have shown that inhibition of AURKA induces polypoidization in mouse models of acute megakaryocytic leukemia.^130^ Mature MKs have also been shown to have reduced expression of AURKA. Interestingly, AURKA activity was found to be elevated in cells with *JAK2*, *CALR* or *MPL* mutations. A recent study showed that MLN8237 (AURKA inhibitor) induced differentiation of *JAK2* and *MPL*-mutated cells as well as decreased BM fibrosis and spleen size in mouse models of MPN and MF, with no obvious myelosupression seen.^131, 132, 133^ Other novel drugs currently in early clinical development for the treatment of PMF include the hedgehog pathway inhibitor PF-0444913, JAK2 specific inhibitors (such as NS-018), and histone deacetylase inhibitors (givinostat, panabinostat and belinostat).^22, 134, 135, 136^ Histone deacetylase inhibitors are promising given the known mutations in PMF that affect the epigenome. Combination therapy with ruxolitinib and other agents are also in development including the histone deacetylase panabinostat, which showed promise in a preclinical trial and there is a current phase I/II trial looking at this combination's benefit in patients with PMF (PRIME trial, clinicaltrials.gov NCT01693601).^137^

## ECM and potential new therapeutics

New therapeutics targeting the ECM may be beneficial in ameliorating the debilitating symptoms due to BM fibrosis. One such novel target might be LOX, considering its above-mentioned influences on BM fibrosis and on promotion of MK proliferation through PDGF-R activation. Another potential novel ECM-based therapy could be targeted against monocyte-derived fibrocytes that may play a role in the pathogenesis of BM fibrosis in PMF. One study showed that neoplastic monocyte-derived fibrocytes were overrepresented in the BM of PMF patients and inhibiting monocytes from differentiating to fibrocytes with recombinant serum amyloid P reduced BM fibrosis in two mouse models of MF.^138^ There is an ongoing phase 2 clinical trial looking at PRM-151 (recombinant serum amyloid P) for the treatment of MF. Preliminary data showed no significant myelosupression and improvement of BM fibrosis in six patients.^139^

Inhibition of cytokines involved in PMF is another potential future ECM-based treatment of PMF. In the GATA-1^low^ mouse model, inhibition of tyrosine kinase of TGF-β1 receptor decreased MF, EMH and neoangiogenesis.^140^ Another potential unexplored treatment in PMF is nintedanib, a multikinase inhibitor that inhibits VEGFR and PDGF receptor. Although bevacizumab (anti-VEGF antibody) was ineffective in treating PMF, inhibition of multiple cytokine receptors involved in the pathogenesis may be efficacious by interrupting cytokine-mediated disruptions of the BM niches and ECM.^141, 142^ TNF inhibitors have shown promise in treating the constitutional symptoms of PMF, although larger trials are necessary to assess safety and efficacy.^143^

## Conclusions and future directions

Considerable advances have been achieved in the understanding of the ECM in BM and its effects on hematopoietic cell biology in health and disease. However, treatment of MF, which is found not only in PMF but also secondary to many hematological diseases, is still a challenge and options are scarce. One reason for this could be the focus of current therapies on controlling MPN cell proliferation, with the expectation that this in turn would result in amelioration of MF. At present, this approach is at best of minor efficacy in early MF and not indicated in cases of severe MF. Specific targeting of ECM dysregulation to prevent and diminish MF may prove the frontline of research and therapy development in PMF with the greatest promise of relieving symptoms and extending life expectancy of patients.

## Figures and Tables

**Table 1:** Changes in the WHO Diagnostic Criteria for PMF

*2008 Criteria for PMF*	*2016 Criteria for pre-PMF*	*2016 Criteria for overt PMF*
*Major criteria*		
MK proliferiferation and atypia with fibrosis (reticulin and/or collagen) or increased marrow cellularity, granulocytic proliferation and decreased erythropoesis in the absence of fibrosis	MK proliferation and atypia without reticulin fibrosis with increased marrow cellularity, granulocytic proliferation and often decreased erythropoesis	MK proliferiferation and atypia with fibrosis (reticulin and/or collagen)
Does not meet WHO criteria for other myeloid neoplasms	Does not meet WHO criteria for other myeloid neoplasms	Does not meet WHO criteria for other myeloid neoplasms
Presence of *JAK2V617F* or other clonal marker or no evidence of reactive fibrosis.	**Presence of JAK2, CALR or MPL mutation** or other clonal marker with no evidence of reactive fibrosis	**Presence of JAK2, CALR or MPL mutation** or other clonal marker with no evidence of reactive fibrosis
*Minor criteria*		
Leukoerythroblastosis	**Leukocytosis (⩾11 × 10**^**9**^**/L)**	Leukoerythroblastosis
Increased serum lactate dehydrogenase	Increased serum lactate dehydrogenase	Increased serum lactate dehydrogenase
Anemia	Anemia (not due to comorbidities)	Anemia (not due to comorbidities)
Palpable splenomegaly	Palpable splenomegaly	Palpable splenomegaly
		**Leukocytosis (⩾11 × 10**^**9**^**/L)**

Abbreviations: MK, megakaryocyte; PMF, primary myelofibrosis.

Changes from the 2008 WHO criteria have been given in bold.
